# Study on Molecular Information Intelligent Diagnosis and Treatment of Bladder Cancer on Pathological Tissue Image

**DOI:** 10.3389/fmed.2022.838182

**Published:** 2022-06-03

**Authors:** Yanfeng Bai, Huogen Wang, Xuesong Wu, Menghan Weng, Qingmei Han, Liming Xu, Han Zhang, Chengdong Chang, Chaohui Jin, Ming Chen, Kunfeng Luo, Xiaodong Teng

**Affiliations:** ^1^Department of Pathology, The First Affiliated Hospital, Zhejiang University School of Medicine, Hangzhou, China; ^2^Hithink RoyalFlush Information Network Co., Ltd., Hangzhou, China; ^3^College of Computer Science and Technology, Zhejiang University, Hangzhou, China

**Keywords:** bladder cancer, molecular information, pathology, deep learning, PD-L1, p53, molecular subtypes

## Abstract

**Background:**

Molecular information about bladder cancer is significant for treatment and prognosis. The immunohistochemistry (IHC) method is widely used to analyze the specific biomarkers to determine molecular subtypes. However, procedures in IHC and plenty of reagents are time and labor-consuming and expensive. This study established a computer-aid diagnosis system for predicting molecular subtypes, p53 status, and programmed death-ligand 1 (PD-L1) status of bladder cancer with pathological images.

**Materials and Methods:**

We collected 119 muscle-invasive bladder cancer (MIBC) patients who underwent radical cystectomy from January 2016 to September 2018. All the pathological sections are scanned into digital whole slide images (WSIs), and the IHC results of adjacent sections were recorded as the label of the corresponding slide. The tumor areas are first segmented, then molecular subtypes, p53 status, and PD-L1 status of those tumor-positive areas would be identified by three independent convolutional neural networks (CNNs). We measured the performance of this system for predicting molecular subtypes, p53 status, and PD-L1 status of bladder cancer with accuracy, sensitivity, and specificity.

**Results:**

For the recognition of molecular subtypes, the accuracy is 0.94, the sensitivity is 1.00, and the specificity is 0.909. For PD-L1 status recognition, the accuracy is 0.897, the sensitivity is 0.875, and the specificity is 0.913. For p53 status recognition, the accuracy is 0.846, the sensitivity is 0.857, and the specificity is 0.750.

**Conclusion:**

Our computer-aided diagnosis system can provide a novel and simple assistant tool to obtain the molecular subtype, PD-L1 status, and p53 status. It can reduce the workload of pathologists and the medical cost.

## Introduction

Bladder cancer is the most common entity of the urinary tract, with an incidence rate of 350,000–380,000 cases being reported per year worldwide (1, 2). Bladder cancer is broadly categorized into non-muscle-invasive bladder cancer (NMIBC) and muscle-invasive bladder cancer (MIBC). NMIBC frequently recurs at an approximate rate of 50–70% and progresses to MIBC at a rate of 15% (3–5). MIBC is a serious and more advanced stage of bladder cancer with a 5-year survival rate of < 50% (6, 7). Bladder cancer kills around 150,000 people a year (8). However, the progress in the diagnosis and treatment of bladder cancer is slow, and there is still no major advance in the clinical treatment of bladder cancer for more than 30 years until the advent of immunotherapy. Neoadjuvant chemotherapy combined with radical cystectomy is the standard treatment for MIBC (9). Up to half of the patients who underwent neoadjuvant chemotherapy can obtain clinically significant therapeutic response (10). However, which patients with MIBC may benefit from neoadjuvant chemotherapy is not identified in the lack of reliable markers for predicting the effectiveness of chemotherapy (11). Therefore, it is hard to choose the optimal individualized treatment strategy for each patient, and there is still a lack of effective and safe alternatives when tumor cells (TCs) show intrinsic or acquired resistance to chemotherapy drugs. With the rapid development of genetic engineering and molecular biology, molecular profiling is expected to resolve this problem and may be a useful tool for the treatment of bladder cancer. There is an urgent need of molecular profiling for the prognosis and effective treatment of bladder cancer.

It has been clinically proven that the use of accurate status of molecular subtype, programmed death-ligand 1 (PD-L1), and p53 typically helps to determine the appropriate therapy and thus improves the survival rate for patients with bladder cancer (12–21). At present, identifying biomarkers for molecular profiling has mainly included the use of immunohistochemistry (IHC) and next-generation sequencing (NGS) and evaluated by manual quantitative evaluation. The work is expensive, time-consuming, and laborious with subjective variations and not adequately used for the diagnosis and treatment of bladder cancer. Although Iwatate et al. (22) had tried to predict the P53 status and PD-L1 status of pancreatic tumors from CT images with radiogenomics, the area under the curve (AUC) values for p53 and PD-L1 predictive models were only 0.795 and 0.683, respectively. Therefore, it is necessary to explore new methods for automatic analysis of the information of tumor molecular.

In recent years, more and more attention has been paid to the application of deep learning in the analysis and processing of pathological images. In Nicolas et al. (23) trained a convolutional neural network (CNN) on histopathology images obtained from The Cancer Genome Atlas (TCGA) to accurately classify whole-slide pathology images into adenocarcinoma, squamous cell carcinoma, or normal lung tissue (24). This method outperforms a pathologist and achieves better sensitivity and specificity. Saltz et al. developed a deep learning method to generate the mapping of tumor-infiltrating lymphocytes (TILs) from standard pathology cancer images, which can help pathologists quickly obtain tumor-immune information (25). In (26) Jacob et al. showed that deep residual learning can predict microsatellite instability (MSI) of gastric adenocarcinoma and colorectal cancer directly from H&E histology, which can provide immunotherapy to a much broader subset of patients with gastrointestinal cancer (27).

In this study, we aimed to establish a computer-aided diagnosis system based on deep learning to predict the molecular subtypes, the PD-L1 status, and the p53 status directly of bladder cancer from pathological images. To complete this system, our work can be divided into five subtasks: (1) segment tumor area from whole slide images (WSIs); (2) train a CNN for molecular subtype recognition; (3) train a CNN for PD-L1 status recognition; (4) train a CNN for p53 status recognition; and (5) evaluate the computer-aided diagnosis system. Four independent CNNs were employed to handle different classification tasks. Due to the outstanding performance of ResNet in alleviating vanishing and exploding-gradient, ResNet was employed as CNNs in this study (28). After the training of four independent CNNs, we develop a computer-aided diagnosis system to automatically predict molecular subtypes and the status of p53 and PD-L1 directly from pathological images. This system can reduce the workload of pathologists and accelerate the identification of molecular subtypes and the status of p53 and PD-L1.

## Data and Methods

### Data Preparation

This study was approved by the Ethics Committee of The First Affiliated Hospital, Zhejiang University School of Medicine with the informed consent waived. A total of 119 patients with MIBC who underwent radical cystectomy from January 2016 to September 2018 at the Department of Pathology, The First Affiliated Hospital, Zhejiang University School of Medicine were enrolled in this study. Pathologic diagnosis and tumor-node-metastasis staging were performed according to the current World Health Organization (29) classification of and the 8th American Joint Committee on Cancer (30). All the pathological sections are scanned into digital WSIs with a 3DHISTECH Pannoramic SCAN. Due to the same molecular information between adjacent sections, IHC results of adjacent sections were recorded as labels of corresponding slides after confirmation by a senior pathologist.

Programmed death-ligand 1 IHC was conducted using the following assay: Ventana/SP263 (Ventana Benchmark Ultra, pre-diluted antibody, pre-treatment: CC1 64 min incubation at 100°C). Human placenta sections were used as positive controls. Monoclonal antibodies against CK5/6 (CK5/6.007, Invitrogen), CK20 (EP23, Invitrogen), CD44 (VFF-7, LabVision), and p53 (D0-7, LabVision) were used according to Envision protocols. Two pathologists blinded to the patients’ outcomes evaluated the results. PD-L1 expressions on TCs and immune cells (ICs) were evaluated. The overall PD-L1 positivity was defined as PD-L1 expressions on ≥ 25% of all TCs and/or ICs. p53 abnormal staining was defined as 0 or > 50% nuclear staining. For the molecular subtype in MIBC, the biomarkers CD44 and CK5/6 score as a surrogate for the basal subtype and CK20 for the luminal subtype. Each case was designated as a basal or luminal subtype using the method based on the highest score of any of the above three markers (31).

### Statistical Analyses

As shown in Table 1, these patients are composed of 103 male and 16 female subjects with a mean age of 70 years (range from 47 to 90 years). For molecular subtype, there were 51 cases of luminal type, 62 cases of basal type, and 6 cases of double-negative type. For PD-L1, there were 40 positive cases and 79 negative cases. For P53, there were 103 wild cases and 16 abnormal cases. Chi-squared test was used to evaluate the relevance between PD-L1 expression, molecular subtype, P53 expression, and the clinicopathological parameters. After statistical analysis, the PD-L1 positivity was significantly associated with age < 70 (*p <* 0.05). Compared with non-basal subtypes, the positive rate of PD-L1 in basal subtypes was higher (*p* < 0.05). There was no significant relationship observed between PD-L1 expression, sex, tumor stage, lymph node status, nerve infiltration, tumor size, and p53 expression.

**TABLE 1:** Clinical features and molecular information of patients with muscle-invasive bladder cancer (MIBC).

Variables		Values
Age (years)		70 (47–90)
Sex	Male (cases)	103
	Female (cases)	16
Molecular subtype	Luminal (cases)	51
	Basal (cases)	62
	Double-negative (cases)	6
PD-L1 status	Positive (cases)	40
	Negative (cases)	79
p53 status	Wild (cases)	103
	Abnormal (cases)	16

### Method

#### Model Construction

**FIGURE 1:**
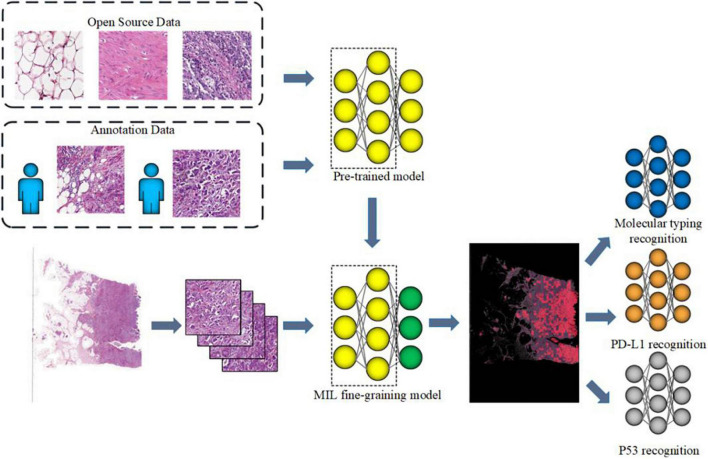
Workflow of muscle-invasive bladder cancer (MIBC) histopathological molecular information recognition with convolutional neural networks (CNNs).

The flowchart of the method is shown in Figure 1. Given a WSI, the tumor areas were first segmented with a weekly supervised framework, then molecular subtypes, p53 status, and PD-L1 status of those tumor areas would be identified by three independent CNNs. Finally, we developed a computer-aided diagnosis system to automatically predict the molecular subtypes, the PD-L1 status, and the p53 status directly from pathological images. As illustrated in Figure 1, the proposed method consists of four parts that include tumor area segmentation, molecular subtype recognition, PD-L1 status recognition, and p53 status recognition.

(1) Tumor area segmentation

Since cancerous urothelial cells have infiltrated into other tissues and are fragmentary in the image, the pixel-level annotation of pathological images is a fallible and time-consuming task. Large number of pathological images with pixel-level annotations were not easily accessible. Inspired by Xu et al. (32), a weekly supervised framework, CAMEL, was employed for tumor area segmentation using only image-level labels. Instead of introducing more supervision constraints, CAMEL splitted the WSI into latticed instances (1,024 × 1,024 pixel tiles at 10 × magnification) and automatically generated their instance-level labels with a combined multiple instance learning (cMIL) approach. After the instance-level dataset was prepared, an instance classifier was trained in a fully supervised manner. The architecture used in both cMIL and the instance classifier was ResNet-34 (28). Then the label of these latticed instances was refined using the trained instance classifier. Finally, the refined instance-level labels were directly assigned to their corresponding pixels. Therefore, a segmentation model based on U-Net (33) with SE block (34) [U-SE-Net (35)] can be fully supervised and trained with the pixel-level labels. The sequeze and excitation (SE) block are added after the Encoder or Decoder block in U-Net. The detail of CAMEL can be found in Xu et al. (32). The dataset was randomly allocated into the training set (49 cases), the validation set (33 cases), and the testing set (37 cases). This model was developed and trained on the training set, and the hyper-parameters were fine-tuned using the validation set. The performance of this model was evaluated on the testing set.

(2) Molecular information recognition

Clinically, the molecular information of MIBC is analyzed by quantifying the expression status of tumor and surrounding cells. Therefore, 39,562 image tiles with 50% or above 50% tumor area were selected from 119 WSIs for the further molecular information recognition. Due to the different distribution in three kinds of molecular information, three independent deep learning models were trained for the recognition of molecular subtypes, PD-L1, and p53 status, respectively. ResNet-50 was employed to implement these models. According to the actual proportion of cases in different molecular information, the dataset was allocated into the training set, the validation set, and the testing set. All models were developed and trained on the training set, and their hyper-parameters were fine-tuned using the validation set. The performance of all models was evaluated on the testing set. The detail of the dataset for molecular information recognition is described in Table 2. It is worth saying that the double-negative type was eliminated for the molecular subtype because this type was relatively rare in our dataset.

**TABLE 2:** The overview of the dataset for molecular information recognition.

Cohorts			Training	Validation	Testing
Molecular subtypes	No. cases	Liminal	18	14	19
		Basal	21	16	25
	No. tiles	Liminal	4890	4720	6962
		Basal	5878	5690	8345
PD-L1 status	No. cases	Positive	18	10	12
		Negative	31	23	25
	No. tiles	Positive	5421	3614	4252
		Negative	10576	7159	8540
p53 Status	No. cases	Wild	40	29	34
		Abnormal	7	4	5
	No. tiles	Wild	13269	9485	11599
		Abnormal	2062	1357	1790

#### Model Training

All models in this study were implemented on Keras v2.2.5 and trained on two NVIDIA GTX 1080Ti GPUs. Keras is a deep learning application programming interface (API) written in Python, running on top of the machine learning platform TensorFlow.

(1) The training of tumor area segmentation: We applied random rotation, random mirroring, and random scaling to augment the training data. Both ResNet-34 in cMIL and the instance classifier were trained using an Adam optimizer with a fixed learning rate of 0.001. In cMIL, ResNet-34 was fine-tuned with the pre-trained model on ImageNet, and the batch size was set to 2. In the instance classifier, the batch size was set to 32. During the segmentation stage, the segmentation model was training using Adam optimizer (36) with a fixed learning rate of 0.001 and the batch size was set to 16. Due to the limitation of the GPU memory, the 1,024 × 1,024 pixel tiles and their corresponding masks were resized to 512 × 512 images.

(2) The training of molecular information recognition: Data augmentation, such as flipping, contrast adjustment, random rotation (rotation angle range 0–180°), random mirroring, and random scaling, was manipulated to avoid overfitting. Each ResNet-50 was fine-tuned with the pre-trained model on ImageNet using Adam optimizer with a warm-up strategy. The learning rate was set to 0.01 and then linearly decreased to 0.001 in 100 epochs. The batch size was set to 32. For molecular subtypes and PD-L1 status recognition, the optimization objective function was computed by the Cross-Entropy Loss Function. For p53 status recognition, in consideration of the extremely imbalanced sample distribution in p53 status, focal loss (37) was employed to deal with the extreme imbalance in the sample distribution of p53 status. Focal loss can be formulated as:


(1) $$\text{FL}\,\left(p_{\text{t}}\right)=-\alpha \,{\left(1-p_{\text{t}}\right)}^{\gamma }\,\log\left(p_{\text{t}}\right)$$

Where *p*_t_ can be defined in formula (2). α is the weight of classt, γ is a parameter to control the weight and generally set to 2. An appropriate α could make imbalance problem solved more easily, we set α to 0.25 in our experiment.


(2) $$p_{t}=\left\{ \begin{array}{c} p\;\mathrm{if}\,y=1\\ 1-p\,\mathrm{otherwise} \end{array}\right.$$

## Results

### Evaluation of Tumor Area Segmentation

**FIGURE 2:**
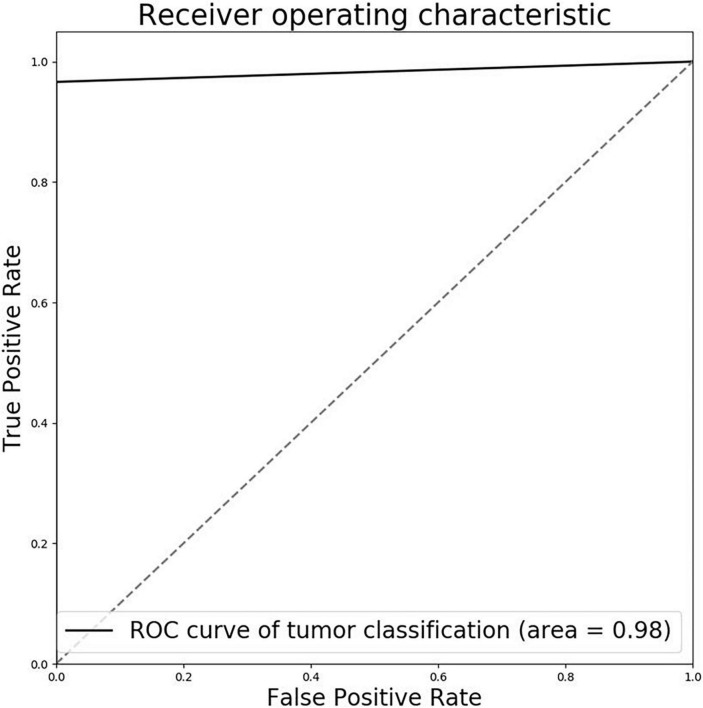
Receiver operator characteristic (ROC) curve of tumor classification pre-trained model.

The testing set was annotated by three experienced pathologists (all with over 10 years of experience in the pathological diagnosis of bladder cancer) and checked by two senior pathologists (both with more than 15 years of experience in the pathological diagnosis of bladder cancer) as the ground truth for evaluation. The instance classifier was evaluated on the testing set, the accuracy and AUC of the model are 0.9958 and 0.98 (Figure 2). Then the instance-level labels of the training set are assigned to the corresponding pixels to generate the pixel-level labels. Therefore, a fully supervised segmentation model can be trained. We have compared the performance of U-Net and U-SE-Net. The performance was quantified by using the Dice score (Dice), pixel accuracy (Pacc), and Jaccard score (Jac) as follows:


(3) $$D\,i\,c\,e=\frac{2\times N_{\text{tp}}}{2\times N_{\text{tp}}+N_{\text{fp}}+N_{\text{fn}}},$$

(4) $$P\,a\,c\,c=\frac{N_{\text{tp}}+N_{t\,n}}{N_{\text{tp}}+N_{t\,n}+N_{\text{fp}}+N_{\text{fn}}},$$

(5) $$J\,a\,c=\frac{N_{\text{tp}}}{N_{\text{tp}}+N_{\text{fp}}+N_{\text{fn}}},$$

where *N_tp_*, *N_tn_*, *N_fp_*, and *N_fn_* are the number of pixels for true positive, true negative, false positive, and false negative.

**FIGURE 3:**
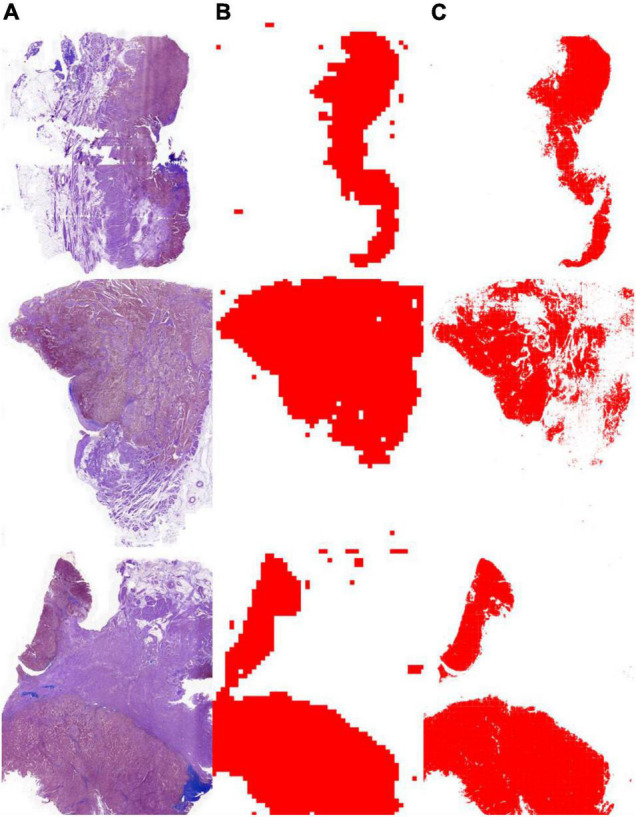
Tumor segmentation results with the instance classifier and U-SE-Net. **(A)** pathological images; **(B)** segmentation results with the instance classifier (red masks); **(C)** segmentation results with U-SE-Net (red masks).

As shown in Table 3, the segmentation performance of the instance classifier, U-Net, and U-SE-Net is listed. From the results in Table 3, we can draw the following conclusions: (1) with the supervision of the instance-level labels, U-Net and U-SE-Net can obtain better segmentation performance; (2) SE blocks in U-SE-Net can exploit adaptive channel-wise feature recalibration to boost the generalization performance, so U-SE-Net outperforms the instance classifier and U-Net. The segmentation results of the instance classifier and U-SE-Net are visualized in Figure 3, which can also prove that the above conclusion is reliable.

**TABLE 3:** The tumor segmentation performance of the instance classifier, U-Net, and U-SE-Net.

Methods	Dice	Paac	Jac
Instance classifier	0.784	0.832	0.796
U-Net	0.894	0.915	0.903
U-SE-Net	0.947	0.952	0.958

### Evaluation of Molecular Information Recognition

**FIGURE 4:**
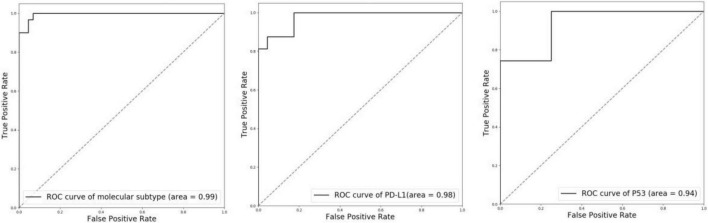
ROC curves of molecular subtype, PD-L1 status, and p53 status with CNN recognition. Left: molecular subtype; Middle: PD-L1 status; Right: p53 status.

To evaluate the performance of molecular information recognition, the result of the testing set is reported in Table 4. The prediction of each case was generated by the average score of the corresponding tumor area tiles. For molecular subtype recognition, the accuracy, sensitivity, and specificity were 0.946, 1.000, and 0.909, respectively. For PD-L1 status recognition, the accuracy, sensitivity, and specificity were 0.897, 0.875, and 0.913, respectively. For p53 status recognition, the accuracy, sensitivity, and specificity were 0.846, 0.857, and 0.750, respectively. The ROC curves of each subtask are shown in Figure 4. To further compare with the machine learning method, the random forest algorithm^1^ was used to select useful features from pathomics features and build the prediction models for molecular information recognition. In total, 154 pathomics features, such as pixel intensity, morphology, and nuclear texture for each ROI, were extracted with the CellProfiler platform (version 2.2.1).^2^ The results in Table 4 indicate that CNNs were superior to the machine learning method.

**TABLE 4:** The performance of molecular information recognition with convolutional neural networks (CNNs) or machine learning.

Methods	Molecular information	Accuracy	Sensitivity	Specificity
CNNs	Molecular subtype	0.946	1.000	0.909
	PD-L1 status	0.897	0.875	0.913
	p53 status	0.846	0.857	0.750
Machine learning	Molecular subtype	0.856	0.871	0.861
	PD-L1 status	0.812	0.820	0.823
	p53 status	0.785	0.778	0.686

## Discussion

Bladder cancer is one of the most common malignant tumors of the urinary system, with approximately 400,000 new cases diagnosed each year. The traditional chemotherapy regimen has limited efficacy and is prone to relapse and metastasis with a poor overall prognosis (38). Unfortunately, there are few advances in its clinical management due to the poor understanding of the correlation between its molecular and clinical features. The molecular biomarkers and pathways involved in bladder cancer are key to understanding its biological heterogeneity and identifying specific subtypes that can be used to predict clinical outcomes and treatment responsiveness to personalized therapies (39). In general, IHC and NGS are often used to identify biomarkers for molecular profiling. However, they are not adequately used in the diagnosis and treatment of bladder cancer because these works are expensive, time-consuming, and laborious with subjective variations. In this study, a computer-aided diagnosis system based on deep learning is established for automatic analysis of the tumor molecular information directly from the pathological image. This computer-aided diagnosis system can be an effective tool to reduce the workload of pathologists.

There is some software available for molecular analysis. However, the available software, such as HALO,^3^ is complex and expensive to use. Our computer-aided diagnosis system is implemented in an end-to-end manner. Postoperative specimens from 119 patients with MIBC were used for the training and tests. A weakly supervised method was adopted to segment tumor area from WSI using only image-level labels. With only image-level labels, our method can segment tumor area with high Dice score, pixel accuracy, and Jaccard score (Dice score: 0.947, pixel accuracy: 0.952, and Jaccard score: 0.958). Then three independent ResNet-50 were trained for the recognition of molecular subtype, PD-L1 status, and p53 status. All ResNet-50 were fine-tuned with the pre-trained model on ImageNet. Focal loss was further introduced to alleviate the extreme imbalance between positive and negative samples in p53 status. For molecular subtype recognition, the accuracy is 0.94, the sensitivity is 1.00, the specificity is 0.909. For PD-L1 status recognition, the accuracy is 0.897, the sensitivity is 0.875, and the specificity is 0.913. For p53 status recognition, the accuracy is 0.846, the sensitivity is 0.857, and the specificity is 0.750. These results verify the effectiveness of our system.

**FIGURE 5:**
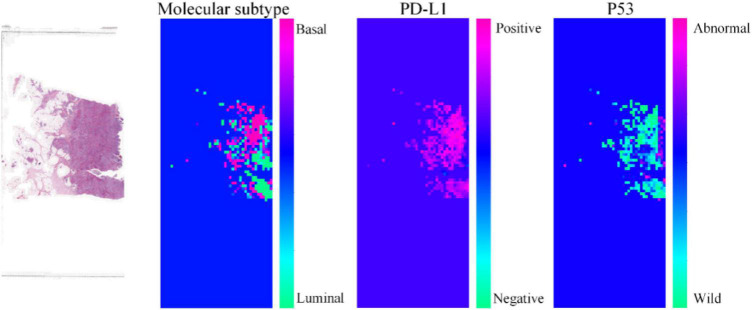
Heatmaps of the same pathological image predicted by our proposed method for molecular subtype, PD-L1 status, and p53 status.

To further demonstrate the effectiveness of our proposed method, we visualize the probability heatmaps in Figure 5. These heatmaps can be used as an IHC result for pathologists to conduct further MIBC research and prove what our proposed method has learned. More comparisons of molecular information interpretation results between IHC and our proposed method can be found in the “Supplementary Material.” The above results confirm that our computer-aided diagnosis system can provide a novel and simple assistant tool to obtain the molecular subtype, PD-L1 status, and p53 status. It can reduce the workload of pathologists and the medical cost.

Although the proposed method achieved excellent performance, this study still had several limitations. First, our proposed method was trained and tested on a relatively small dataset from a single large hospital, lacking multi-center or external data validation. Future research is needed to incorporate more data from multi-hospital. Second, this study was retrospective, and more prospective data are expected to be included in this study in the future. Third, without the pixel-level labels in training data, the performance of tumor segmentation is not ideal and needs to be improved. In addition, finally, the double-negative type was eliminated for the molecular subtype in this study. We hope we can collect more datasets to implement the recognition of the double-negative. Therefore, these limitations will be overcome in our future work.

## Conclusion

This study developed a computer-aided diagnosis system that achieved high performance in the recognition of molecular subtypes, PD-L1 status, and p53 status directly from pathological images based on deep learning. Our computer-aided diagnosis system can provide a novel and simple assistant tool to obtain the molecular subtype, PD-L1 status, and p53 status. It can reduce the workload of pathologists and the medical cost.

## Data Availability Statement

The raw data supporting the conclusions of this manuscript will be made public by the author after approval by the Ethics Committee.

## Author Contributions

YB, HW, and XT: study conception and design. YB, XW, MW, QH, LX, HZ, and CC: acquisition of data. YB, HW, CJ, and MC: analysis and interpretation of data. YB and HW: drafting of the manuscript. HW, CJ, MC, and XT: critical revision of the manuscript for important intellectual content. YB, XW, MW, and KL: statistical analysis. HW: administrative, technical, or material support. XT: supervision. All authors were involved in writing the manuscript and had final approval of the submitted and published versions.

## Conflict of Interest

HW, CJ, MC, and KL were employed by Hithink RoyalFlush Information Network Co., Ltd. The remaining authors declare that the research was conducted in the absence of any commercial or financial relationships that could be construed as a potential conflict of interest.

## Publisher’s Note

All claims expressed in this article are solely those of the authors and do not necessarily represent those of their affiliated organizations, or those of the publisher, the editors and the reviewers. Any product that may be evaluated in this article, or claim that may be made by its manufacturer, is not guaranteed or endorsed by the publisher.

## Footnotes

1
https://scikit-learn.org/stable/modules/ensemble.html#forest

2
https://cellprofiler.org/

3
https://www.indicalab.com/halo
