# Supplementary material for: Study on Molecular Information Intelligent Diagnosis and Treatment of Bladder Cancer on Pathological Tissue Image
Source: Front Med (Lausanne). 2022 Jun 3;9:838182. doi: 10.3389/fmed.2022.838182 (PMC9215327; doi:10.3389/fmed.2022.838182)
Supplement: Supplementary file 1 [file Presentation_1.pdf]

Study on molecular information intelligent diagnosis and treatment of bladder cancer on pathological tissue image

Yanfeng Bai[1*], Huogen Wang[2,3*], Xuesong Wu[1], Menghan Weng[1], Qingmei Han[1], Liming Xu[1], Han Zhang[1], Chengdong Chang[1], Chaohui Jin[2], Ming Chen[2], Kunfeng Luo[2], Xiaodong Teng[1]

[1]Department of Pathology, The First Affiliated Hospital, Zhejiang University School of Medicine, Hangzhou 310003, Zhejiang, China

[2]Hithink RoyalFlush Information Network Co., Ltd, No. 18 Tongshun Street, Hangzhou 310012, Zhejiang, China

[3]College of Computer Science and Technology, Zhejiang University, Hangzhou 310013, Zhejiang, China

Corresponding author: Xiaodong Teng, Department of Pathology, The First Affiliated Hospital, Zhejiang University School of Medicine, Hangzhou 310003, Zhejiang, China. Phone:86-571-8723-6368; Fax: 86-571-8723-6368; Email: teng1102069@zju.edu.cn

Keyword: Bladder cancer; Molecular information; Pathology; Deep learning; PD-L1; p53; molecular subtypes

In this supplementary material, the comparisons of molecular information interpretation results between IHC and our proposed method are shown.

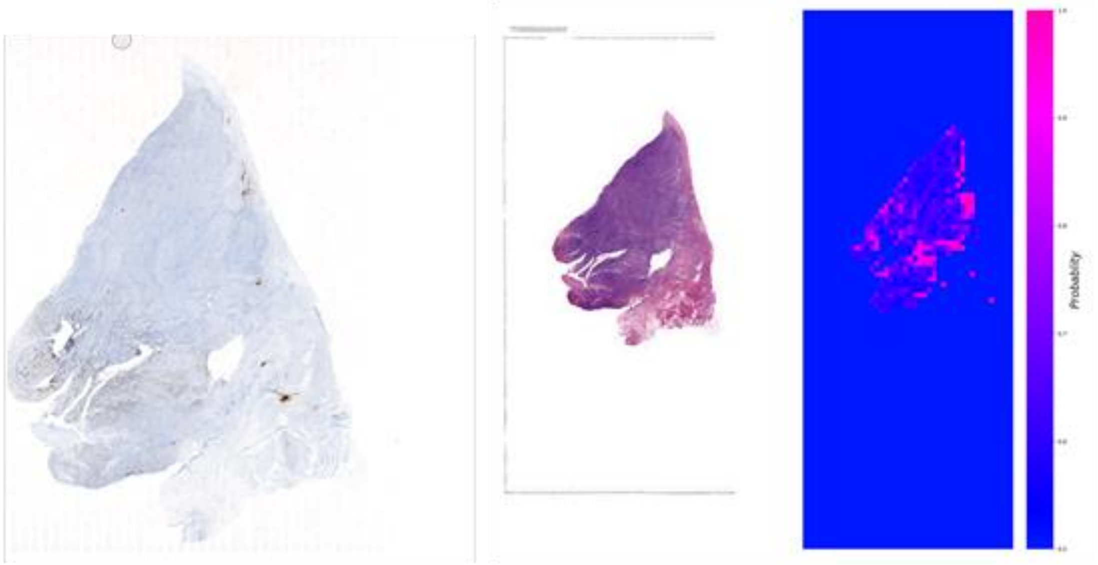

(a) p53 55%, IHC: abnormal type, DCNN: abnormal type.

[*] The authors contribute equally.

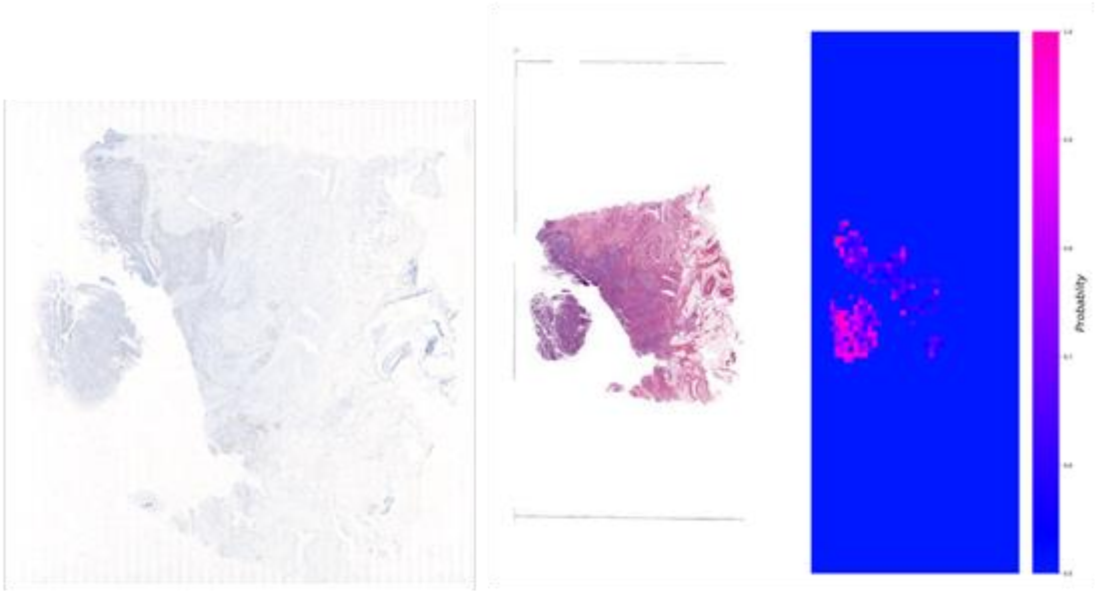

(b) p53 20%, IHC: wild type, DCNN: wild type.

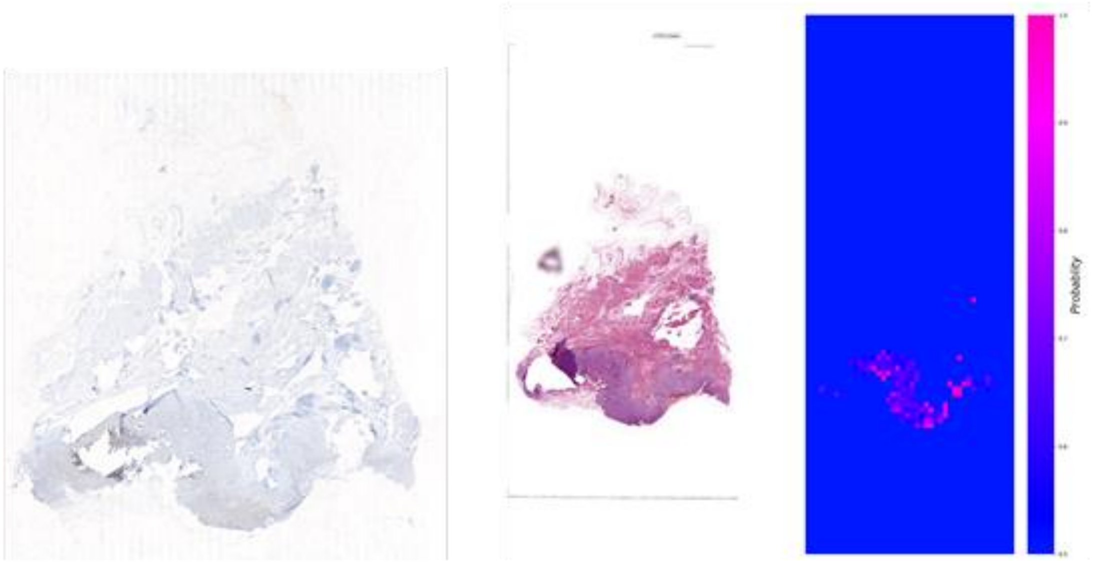

(c) p53 60%, IHC: abnormal type, DCNN: abnormal type.

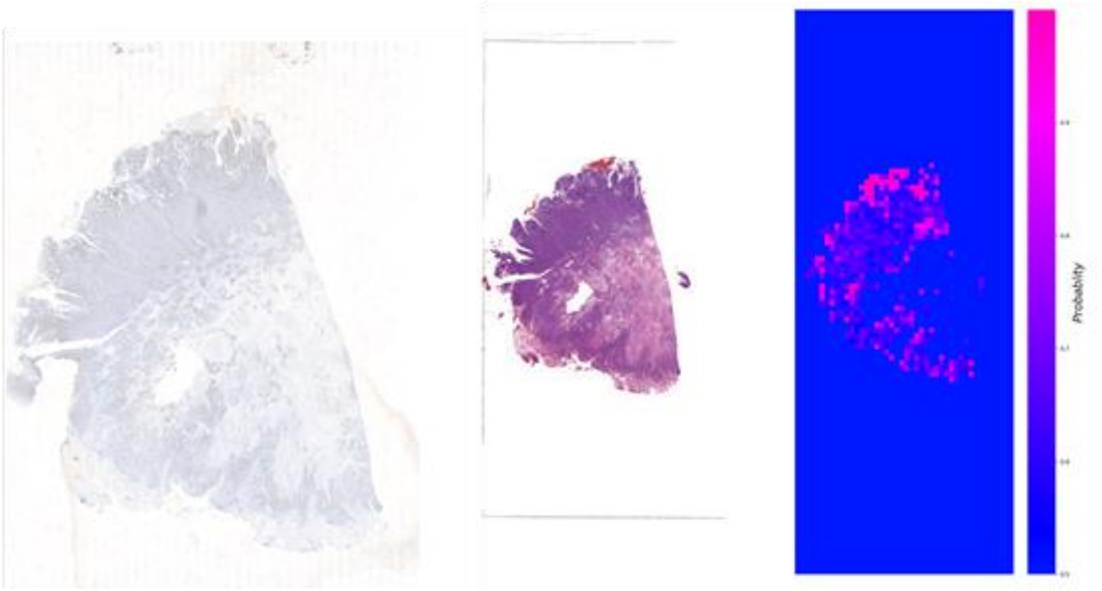

(d) p53 55%, IHC: abnormal type, DCNN: abnormal type.

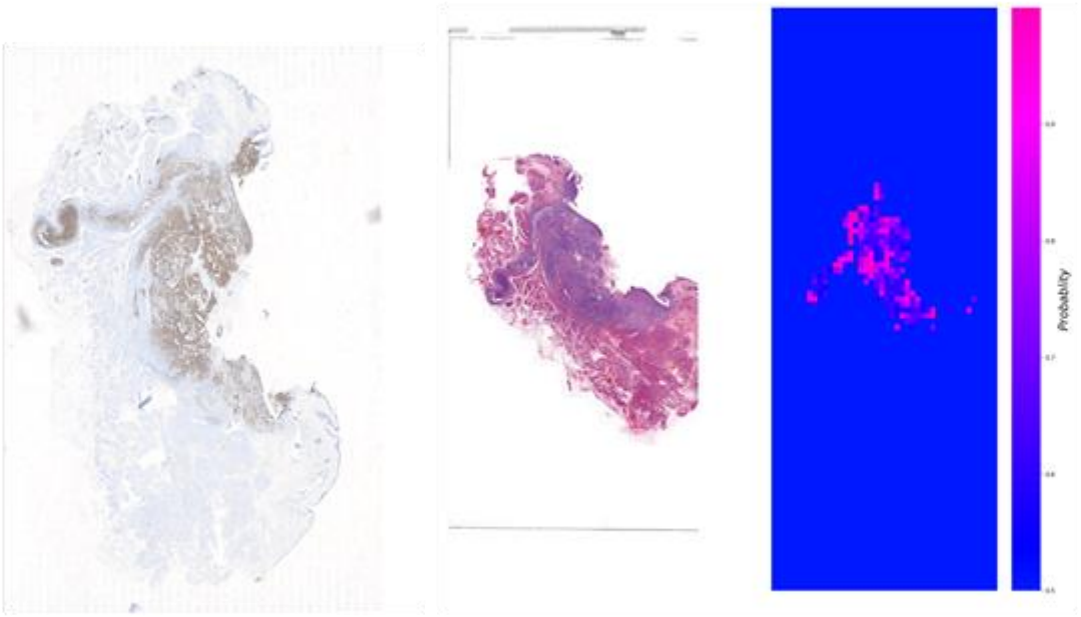

(e) p53 80%, IHC: abnormal type, DCNN: abnormal type.

Figure 1. IHC results of P53 (left) and P53 abnormal type probablity heatmaps predict by DCNN with H&E stained images (right).

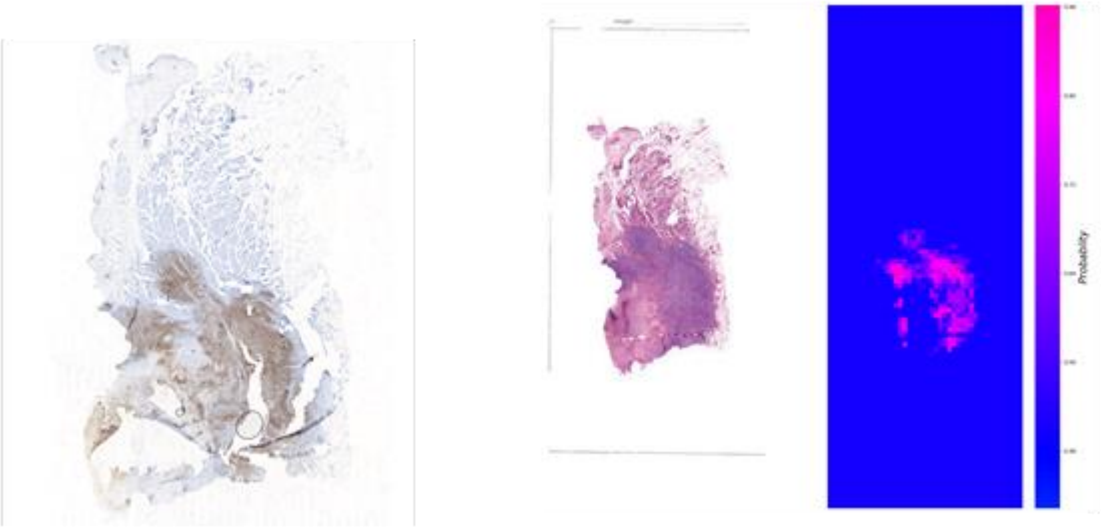

(a) TC 80%, IC 25%, IHC: PDL1 positive type, DCNN: positive type.

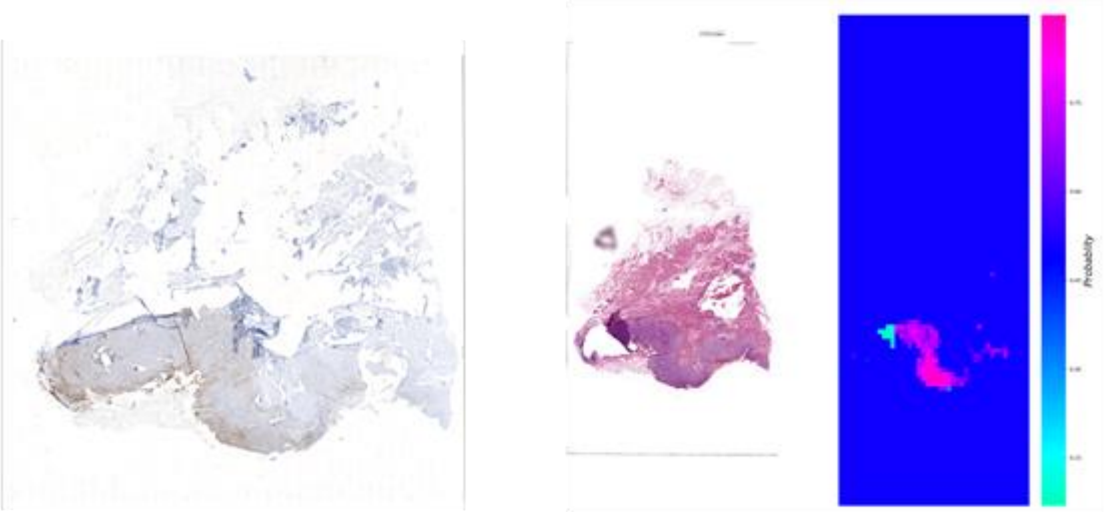

(b) TC 25%, IC 10%, IHC: PDL1 positive type, DCNN: positive type.

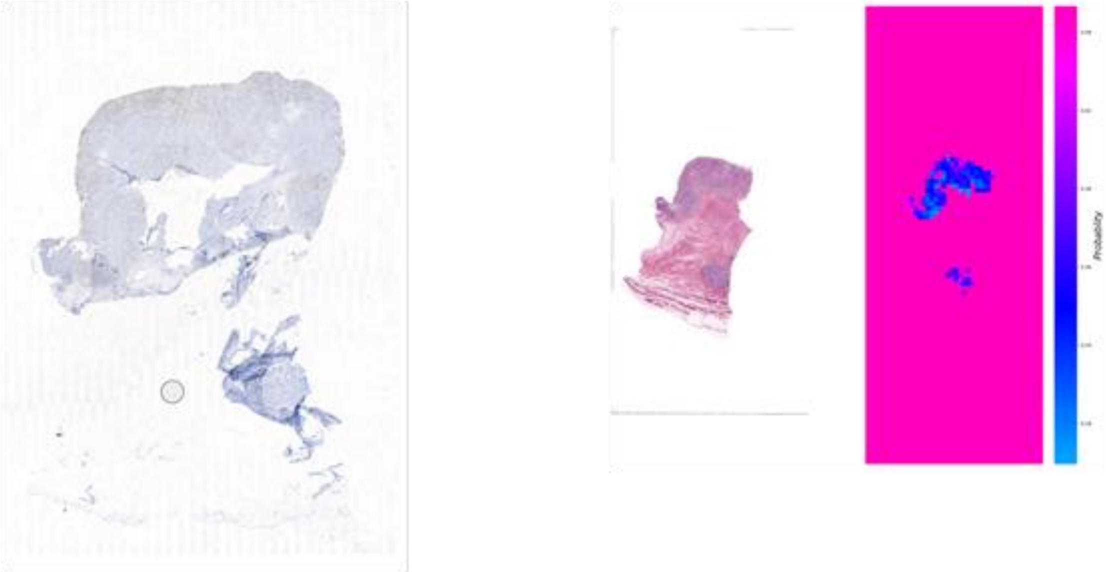

(c) TC 10%, IC 10%, IHC: PDL1 negative type, DCNN: negative type.

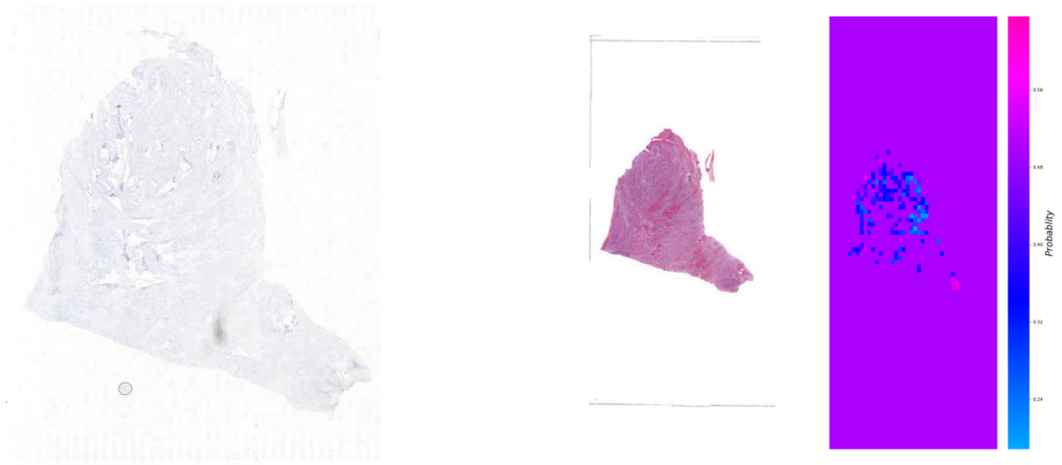

(d) TC 0%, IC 0%, IHC: PDL1 negative type, DCNN: negative type.

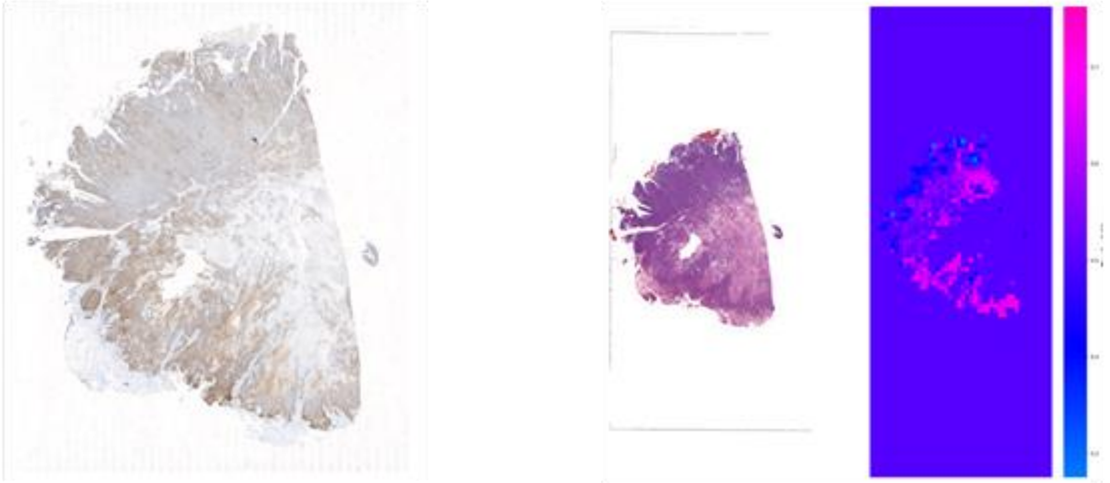

(e) TC 90%, IC 25%, IHC: PDL1 positive type, DCNN: positive type.

Figure 2. IHC results of PDL1 (left) and PDL1 positive probablity heatmaps predict by DCNN with H&E stained images (right).

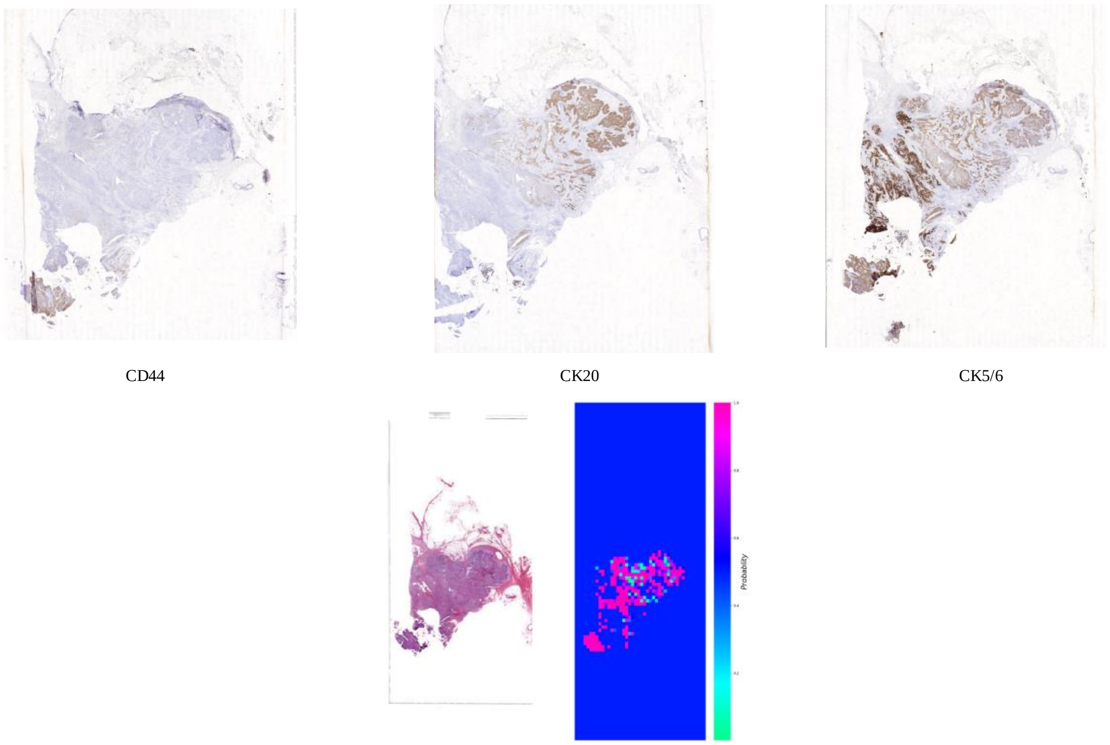

(a) CD44 10%, CK20 0%, CK5/6 95%, IHC: basal type, DCNN: basal type.

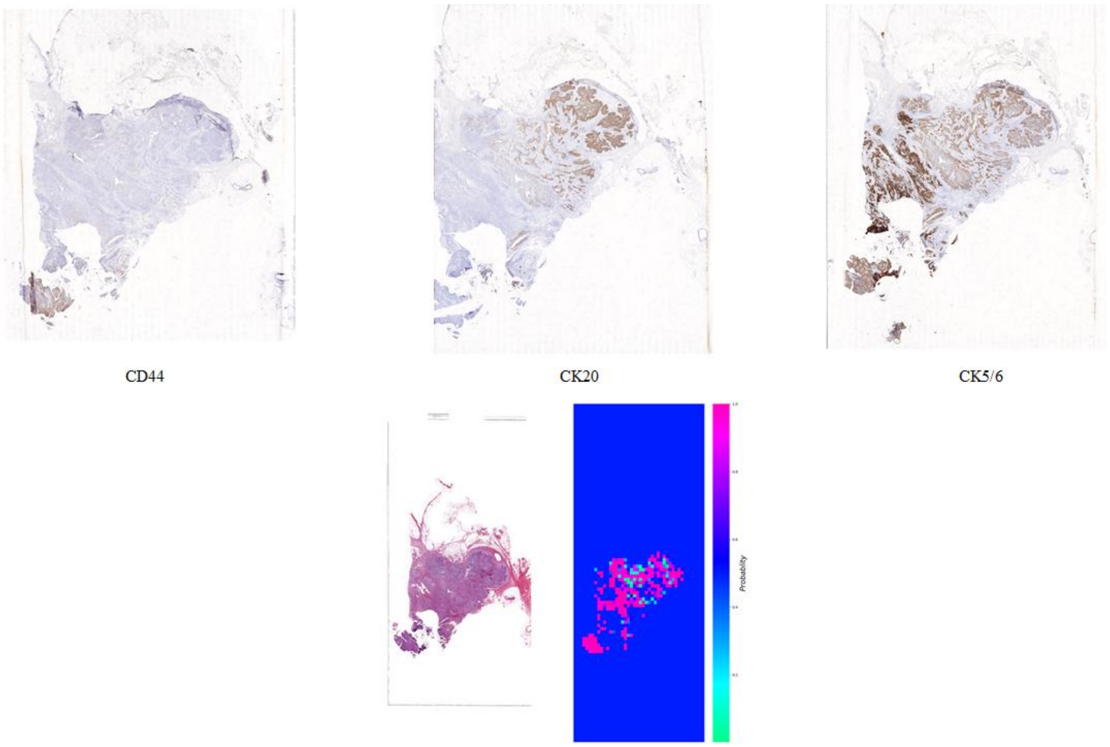

(b) CD44 90%, CK20 0%, CK5/6 0%, IHC: basal type, DCNN: basal type.

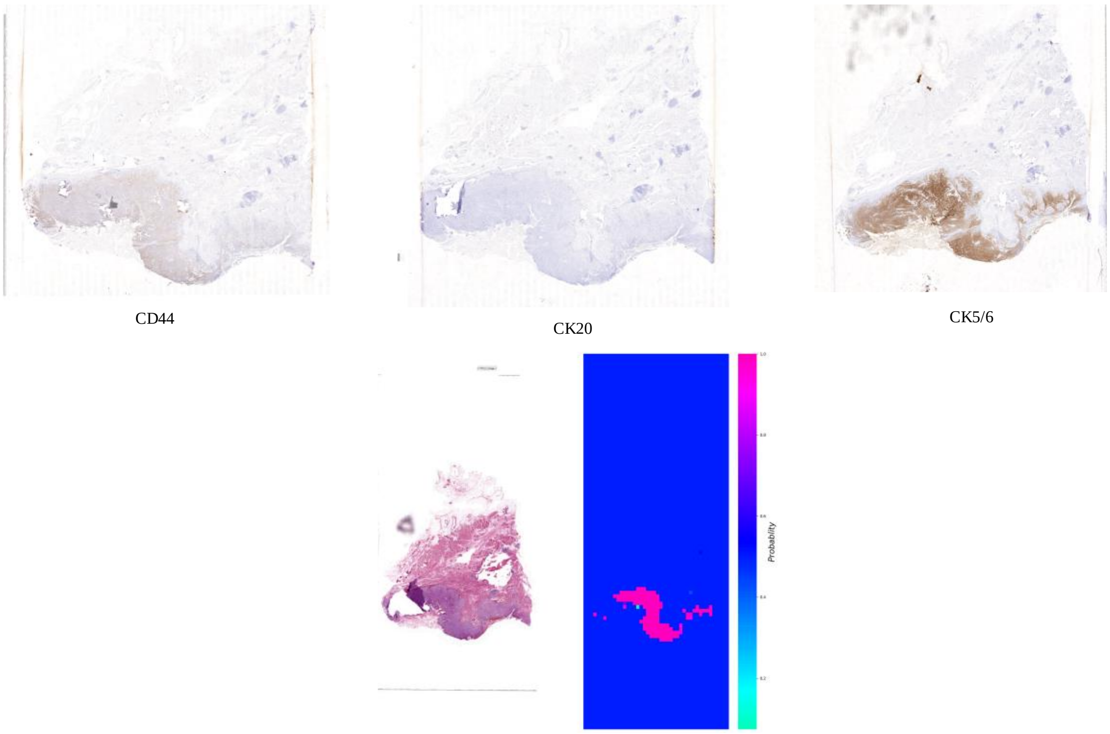

(c) CD44 20%, CK20 0%, CK5/6 95%, IHC: basal type, DCNN: basal type.

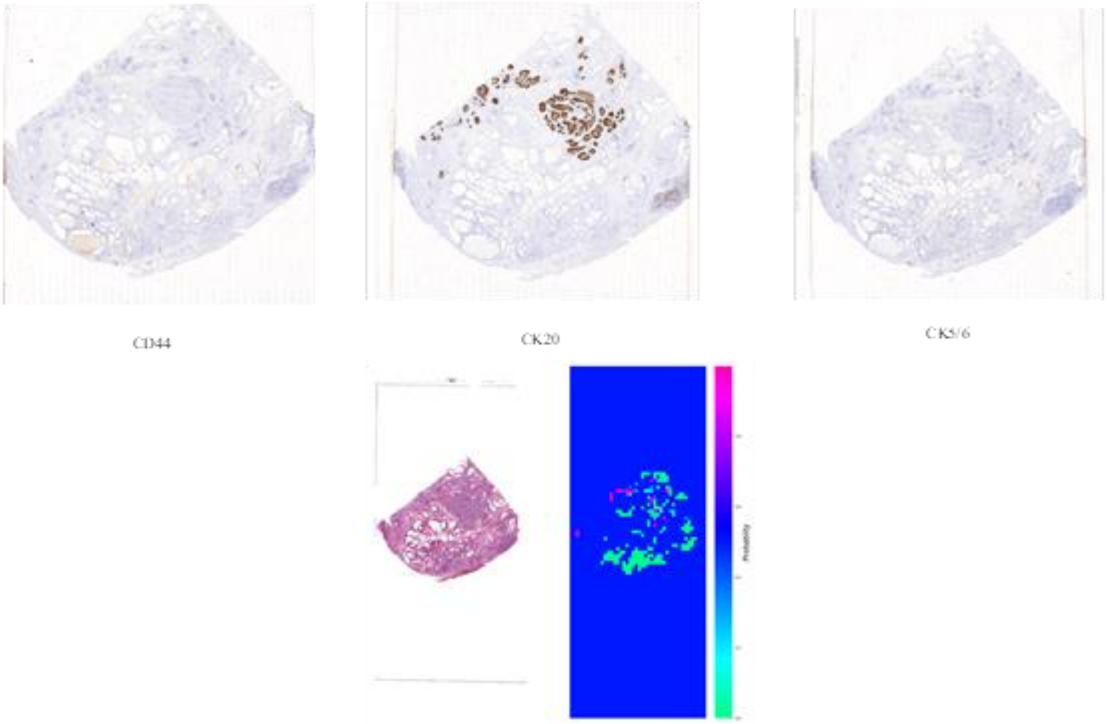

(d) CD44 0%, CK20 85%, CK5/6 0%, IHC: luminal type, DCNN: luminal type.

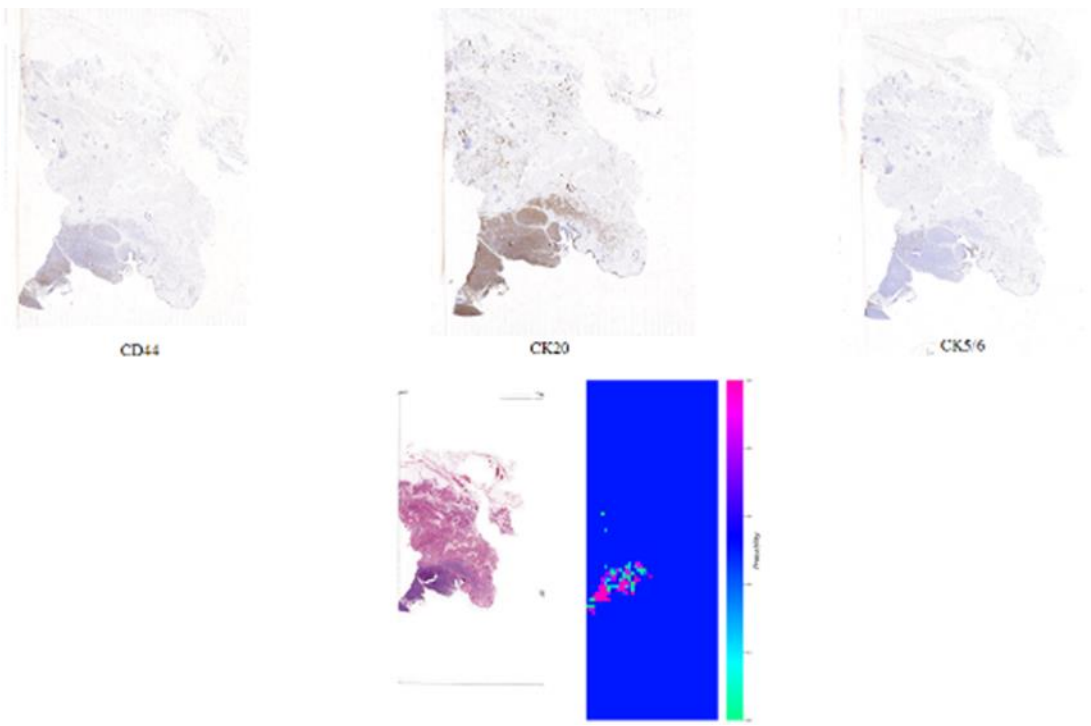

(e) CD44 15%, CK20 95%, CK5/6 5%, IHC: luminal type, DCNN: luminal type.

Figure 3. IHC results of CD44, CK20 and CK5/6 (top three) and molecular subtype probablity heatmaps predict by DCNN with H&E stained images (bottom). In the heatmap, the color of pixel closer with red means the H&E area is predicted with high probablity of luminal stbtype, more green means the area with high probablity of basal subtype.

Some examples with the ground-truth for tumor segmentation in the testing set was shown.

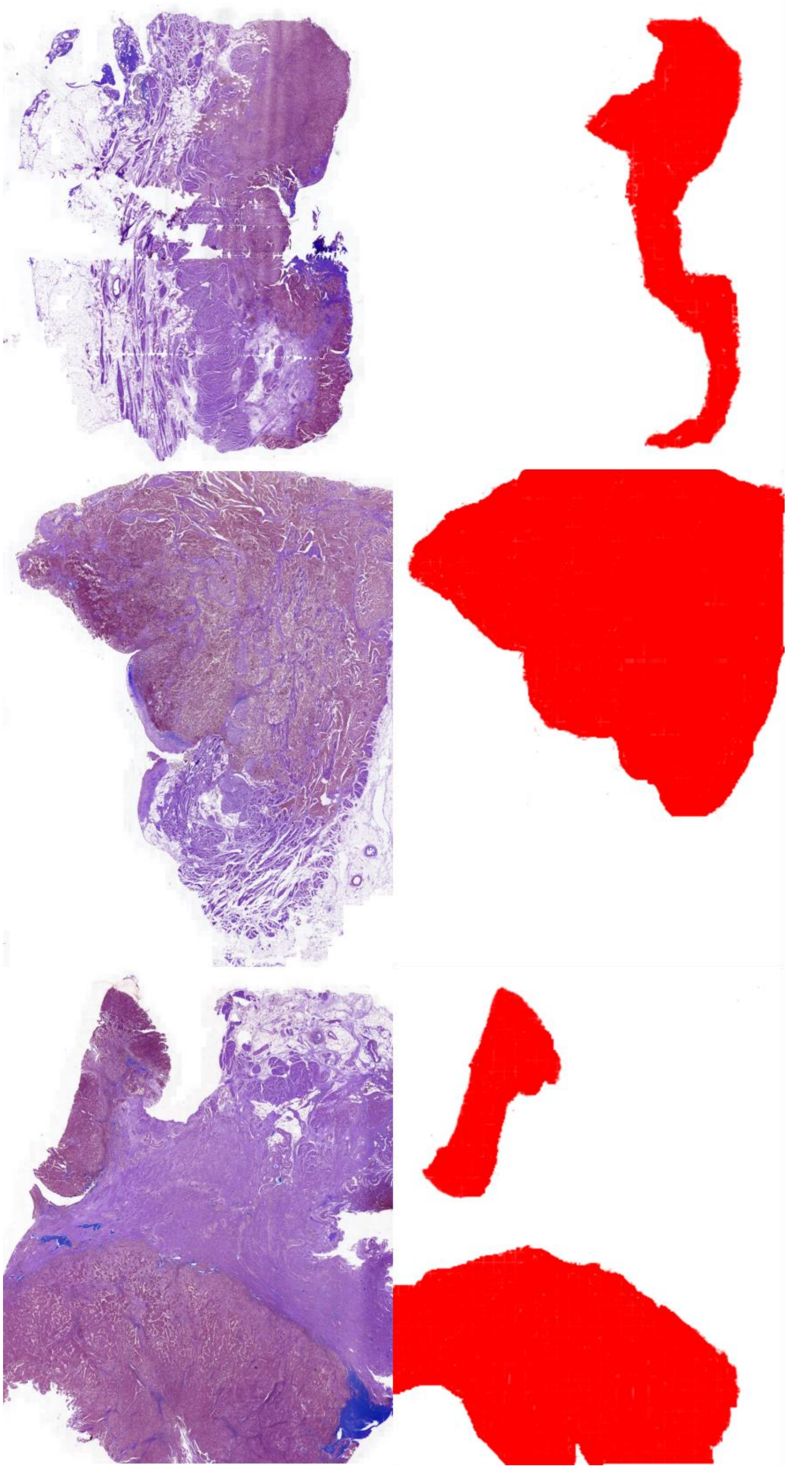

A B

(f) Some examples with the ground-truth for tumor segmentation in the testing set. A: pathological images; B: the ground-truth for tumor segmentation (red masks).

.
